# Accuracy of Electrocardiography and Agreement with Echocardiography in the Diagnosis of Pediatric Left Atrial Enlargement

**DOI:** 10.1038/s41598-020-66987-7

**Published:** 2020-06-22

**Authors:** Charis Ng, Attila Ahmad, Dalton R. Budhram, Mu He, Narayanaswamy Balakrishnan, Tapas Mondal

**Affiliations:** 1 Candidate at Faculty of Medicine, University of Toronto, Toronto, Canada; 2 Department of Pediatric Cardiology, University of Alberta, Edmonton, Canada; 3 Candidate at Faculty of Medicine, Queen’s University, Kingston, Canada; 4 Department of Mathematics & Statistics, McMaster University, Hamilton, Canada; 5 Department of Mathematics & Statistics, McMaster University, Hamilton, Canada; 6 Department of Pediatric Cardiology, McMaster University, Hamilton, Canada

**Keywords:** Cardiology, Paediatric research

## Abstract

Left atrial enlargement (LAE) is a marker for diastolic cardiac dysfunction. Echocardiograms are considered the gold-standard for diagnosis, but given their wider access and lower economic cost, electrocardiograms (ECGs) may be useful in identifying patients who would benefit from further investigation. This study investigates the utility of ECG criteria to diagnose LAE in pediatric patients. A retrospective chart review (n = 492) was conducted in patients whose echocardiograms demonstrated LAE by left atrial indexed diameter z-score ≥2.0 and/or increased left atrial to aortic root ratio at various cutoffs (≥1.4, ≥1.6, ≥1.8). ECG criteria studied included: (1) P wave ≥110 msec, (2) P mitrale ≥40 msec, in LII (3) terminal negative P wave deflection in lead V1 > 40 msec, and (4) P/PR segment >1.6 in lead II. Sensitivity, specificity, Cohen’s Kappa coefficient (κ), and ROC curves were calculated. A combination of P mitrale ≥40 msec and terminal negative P wave deflection in lead V1 > 40 msec yielded the greatest agreement (κ = 0.221, 95%CI 0.060–0.382), but all ECG criteria used to diagnose LAE had poor diagnostic value (AUC < 0.60). The present ECG criteria should not be used to diagnose LAE in the absence of an echocardiogram and findings should be considered in the context of clinical symptoms.

## Introduction

Left atrial enlargement (LAE) has been established as a marker for diastolic dysfunction in left-sided cardiac disease, and a surrogate measurement for conditions including patent ductus arteriosus, ventricular septal defects, mitral valve heart disease, and atrial fibrillation^1,2^. In the pediatric population, these conditions can be associated with significant morbidity and mortality in the absence of early intervention, thus necessitating the development of a reliable and standardized method of diagnosing LAE.

Currently, the gold-standard method for diagnosis is the echocardiogram^3^. In current clinical practice, pediatric patients who are suspicious for LAE often receive both echocardiograms and electrocardiograms (ECG) concurrently. In this method, ECGs are used only to exclude the presence of other cardiological diseases. However, given the comparatively wider access and lower economic cost of ECGs compared to echocardiography, it has been hypothesized that ECGs can be used as a screening test to identify patients who would benefit from further investigation. The development of a standardized guideline for LAE diagnosis using ECGs would help inform the decisions of physicians in rural or developing regions regarding referral to larger centers for specialized pediatric echocardiography. Additionally, identification of patients who do not benefit from further echocardiographic testing would minimize patient risks associated with unnecessary medical testing, decrease wait-times, and allow for a more efficient use of healthcare resources.

A targeted literature search was conducted in order to identify studies assessing ECG diagnostic accuracy for LAE in the pediatric population. In this search, we identified only four papers, all of which were published between 1967 and 1984^4–7^. Of the identified studies, only Biancaniello *et al*. and Maok & Krongrad compare the accuracy of the ECG with echocardiograms. A significant limitation in these studies included their small sample sizes of 52 and 90 children, respectively. Both studies suggested that ECG criteria were moderately predictive, but more sensitive ECG criteria should be developed. The majority of the recent literature regarding LAE diagnosis using ECGs is studied in the context of adults or young athletes as opposed to the pediatric population. Commonly studied criteria include: P mitrale (a notched P wave in lead II ≥120 msec or a notch of ≥40 msec), P wave ≥110, and P axis <30^8–10^. Although some studies have suggested that P wave duration may increase with age, there are no current guidelines that propose appropriate adjustments in these criteria for young infants^11,12^. Given the significant changes in ECG diagnostic criteria in the last 30 years, there is significant value in conducting the first assessment of newer ECG criteria in the context of pediatric LAE. Using the aforementioned criteria from studies focused on adults and young athletes, we conducted the largest and most comprehensive study in a pediatric population to date. In the current study, we assess the use of ECGs for diagnosis of LAE in terms of sensitivity, specificity, and agreement of echocardiograms and ECGs in order to minimize patient risk and optimize the use of healthcare resources.

## Objectives

The aim of this study was to determine the agreement (κ) between echocardiography and one or more combinations of ECG criteria for diagnosis of LAE in pediatric patients (aged 0–18). We also sought to identify whether single or combinations of ECG criterion had sufficient sensitivity and specificity to corroborate echocardiographic findings. In a secondary analysis, we aim to assess the impact of age within the pediatric population on the performance of ECG criteria, with a particular focus on P wave duration.

## Methods

No procedures were performed in this study and all methods were performed in accordance with relevant guidelines and regulations. Formal Hamilton Health Sciences Research Ethics Board (HIREB) approval was received prior to the collection of data for this retrospective study. Due to the retrospective nature of this study, obtaining informed consent was not applicable and an exemption was obtained from HIREB.

### Setting

A retrospective chart review was conducted at McMaster Children’s Hospital (MUMC) over a three-year period from 2013 to 2015 to identify the prevalence of various ECG criteria in pediatric patients with echocardiographically-proven LAE. Participants included children between the ages of 0 and 18 years for whom pediatric cardiologists at MUMC have reported LAE based on echocardiographic data and who received an ECG within two weeks of the echocardiogram. Exclusion criteria included the presence of atrial fibrillation, other arrhythmias at the time of study, presence of a permanent pacemaker, or poor test quality.

### Echocardiogram

A review of echocardiograms for study participants was conducted. Participants with echocardiograms demonstrating one or more of the following criteria were considered to have echocardiographically-proven LAE and were included in the study: left atrial indexed diameter z-score ≥2.0, and elevated left atrial to aortic root (LA/AO) ratio. The ratio was not used if there was any aortic abnormality. Given the grading of LA/AO elevation, analyses were conducted at several cut-offs which included 1.4, 1.6, and 1.8. Criteria are specified in Table 1.

**Table 1:** Participants who met one or more of the echocardiographic criteria were included in this study. Concurrent ECGs were assessed for these ECG criteria.

Echo Criteria		ECG Criteria	
Left atrial indexed diameter z-score (EC1)	≥2.0	P wave duration (C1)	≥110 msec
LA/AO ratio* (EC2)	≥1.4, ≥1.6, ≥1.8	P mitrale (C2)	≥40 msec
		Terminal negative P wave in lead V1 (C3)	≥40 msec
		P/PR segment (C4)	>1.6

*EC1 and EC2 refer to Echo Criteria 1 and Echo Criteria 2*

*C1, C2, C3, C4 refer to ECG Criteria 1, 2, 3, and 4*

*Various LA/AO ratio cut-offs were utilized in the statistical analyses in order to determine optimal values.

### Measurement of electrocardiogram

Standard 12 lead ECGs were retrieved for patients meeting the study criteria. The presence or absence of the following ECG criteria were then evaluated and recorded: 1) a P wave ≥110 msec in lead II, 2) P wave with two peaks separated by a duration of ≥40 msec in lead II (P mitrale), and 3) a terminal negative portion of P wave in lead V1 (the P terminal force) >40 msec, 4) a P/PR segment >1.6 in lead II^8–10^. Of note, two of these criteria have not previously been compared to gold-standard echocardiography in the pediatric population: P mitrale and increased P/PR segment ratio. The ECGs were independently reviewed by two investigators manually from edge to edge using a caliper. A consensus was made if readings differ. Reviewers measuring ECGs were blinded to echocardiogram results. ECG criteria are specified in Table 1.

## Statistical Analysis

Cohen’s Kappa coefficient (κ) were calculated between echocardiographic LAE diagnoses and positive ECG criteria in order to measure agreement between the tests. κ agreements are classified in accordance with the benchmarks proposed in the literature^13^. Sensitivity and specificity were calculated for all ECG indices. Statistical analyses were conducted using R statistical software version 3.4.3 (R Foundation, Vienna, Austria). Receiver operating characteristic (ROC) curves were constructed to investigate the use of single-criterion diagnostic utility against independent echocardiographic criterion.

In very young children with LAE, the aforementioned ECG thresholds may perform differently as compared to older children. To ascertain the impact of age on the utility of these ECG criteria, a post-hoc subgroup analysis was carried out. κ agreement between the tests of all ECG indices were calculated for patients aged <1 year compared with all other patients.

## Results

The records of all pediatric patients seen at McMaster University Medical Centre between 2013 and 2015 for echocardiographic investigations were reviewed. A total of 624 patients were identified in which an ECG was conducted within 2 weeks of an echocardiogram positive for LAE. Of these, 132 patients were excluded from our study due to arrhythmias or poor ECG test quality that prevented an accurate assessment for ECG criteria. The study population ranged from newborn to 18 years of age at the time of their echocardiogram. The patient characteristics are displayed in Table 2. The most commonly diagnosed heart condition is left-to-right shunt, followed by vascular heart disease.

**Table 2:** Patient characteristics.

	*Criteria 1*	Criteria 2*		
	*Left atrial indexed diameter z-score* ≥*2.0 (n* = *113)*	*LA/AO* ≥*1.4 (n* = *475)*	*LA/AO* ≥*1.6 (n* = *207)*	*LA/AO* ≥*1.8 (n* = *74)*
***Sex***				
Female (%)	52 (46%)	248 (52%)	119 (57%)	47 (64%)
Male (%)	61 (54%)	227 (48%)	88 (43%)	27 (36%)
Mean Age in Years ($\pm SD$)	3.9 (4.6)	3.1 (4.5)	2.2 (3.7)	1.8 (3.4)
Height in centimeters ($\pm SD$)	111.5 (33.5)	110.6 (34.8)	106.1 (34.2)	102.7 (35.2)
Weight in kilograms ($\pm SD$)	18.8 (23.4)	15.1 (19.5)	11.2 (34.2)	9.2 (35.2)
Body Mass Index - BMI ($\pm SD$)	18.3 (6.3)	19.1 (7.6)	16.6 (4.1)	16.8 (4.2)
Body Surface Area - BSA ($\pm SD$)	0.65 (0.49)	0.55 (0.46)	0.46 (0.37)	0.40 (0.33)
***Diagnosed Heart Conditions***				
None	2	48	12	2
Tetralogy of Fallot	4	5	2	0
Left-to-right shunt	55	251	123	53
Valvular Heart Disease	51	195	89	30
Ventricular Enlargement	37	87	41	17
Other	36	129	64	30

*Various criteria 2 (LA/AO ratio) cut-offs were utilized in the statistical analyses in order to determine optimal values.

**Figure 1:**
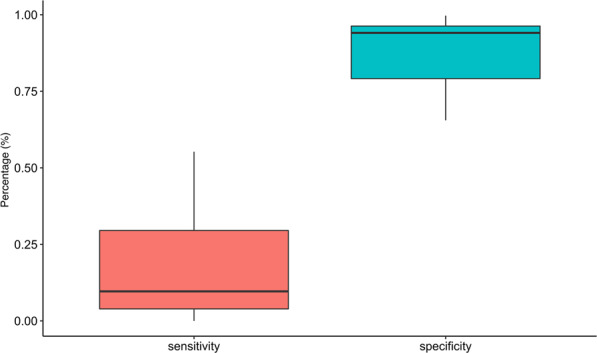
Box Plot summarizing the sensitivity and specificity for all electrocardiogram and echocardiogram criteria combinations (EC2 = 1.8).

The sensitivity, specificity, and measurement of agreement between independent and combinations of ECG criterion were calculated against (1) left atrial indexed diameter z-score ≥2.0, (2) elevated left atrial to aortic root (LA/AO) ratio (1.4, 1.6, and 1.8), and (3) both echocardiogram criteria. The findings for the most significant ECG criteria for diagnosis of LAE are summarized in Table 3. In terms of agreement, a terminal negative p-wave in lead V1 had the greatest agreement (slight-fair) when compared to the presence of both echocardiogram criteria. When this ECG criterion was combined with the presence of a P mitrale, the agreement with LA/AO > 1.8 is optimized (κ = 0.221, CI 0.060–0.382). In general, the diagnostic agreement between echocardiogram and ECG increases for increasing LA/AO ratio cutoffs. The specificity for most criteria combinations is generally high, while the sensitivity is generally low (Fig. 1). In particular, Table 3 demonstrates that the ECG criteria that show the highest agreement with echocardiogram criteria have low-moderate sensitivity (20.8–55.0%) with moderate-high specificity (73.6–96.1%).

**Table 3:** Kappa Values, Sensitivity, and Specificity Values for Most Significant Echo and ECG Criteria grouped by LA/AO Ratio Threshold.

EC2	Criteria	Kappa (95% CI)	Judgments	P-Value	Sensitivity (95% CI)	Specificity (95% CI)
≥1.8	C3 & EC2 **≥** 1.8	0.138* (0.018–0.258)	Slight Agreement	0.0155	0.376 (0.274–0.488)	0.784 (0.741–0.823)
	C2,C3 and EC2 ≥ 1.8	0.221* (0.060–0.382)	Fair Agreement	0.0205	0.208 (0.108–0.341)	0.961 (0.934–0.980)
	C3 and EC1, EC2 ≥ 1.8	0.204* (0.086–0.322)	Fair Agreement	0.0041	0.550 (0.385–0.707)	0.783 (0.735–0.825)
≥1.6	C3 and EC1, EC2 ≥ 1.6	0.125* (0.012–0.238)	Slight Agreement	0.0379	0.403 (0.289–0.525)	0.736 (0.676–0.790)

*All Kappa values are significant (p < 0.05) as calculated by McNemar’s Test.

Judgments refer to the strength of the Kappa Statistic: Slight Agreement (κ = 0–0.20); Fair Agreement (κ = 0.21–0.40).

EC1: Left atrial indexed diameter z-score ≥2.0.

EC2: LA/AO ratio ≥1.6 or ≥1.8.

C2: P mitrale ≥40 msec.

C3: Terminal negative P wave in lead V1 ≥40 msec.

**Figure 2:**
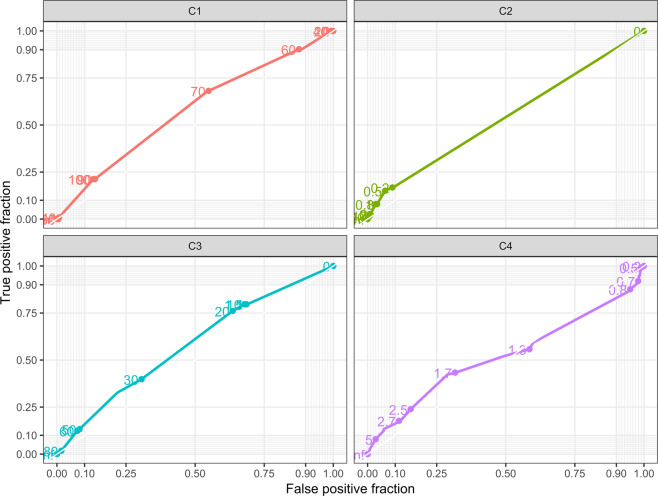
ROC curve for Echocardiographic Criteria left atrial indexed diameter z-score and ECG criteria independent of LA/AO ratio C1: P wave duration ≥ 110 msec C2: P mitrale ≥ 40 msec C3: Terminal negative P wave in lead V1 ≥ 40 msec C4: P/PR segment ≥ 1.6.

**Figure 3:**
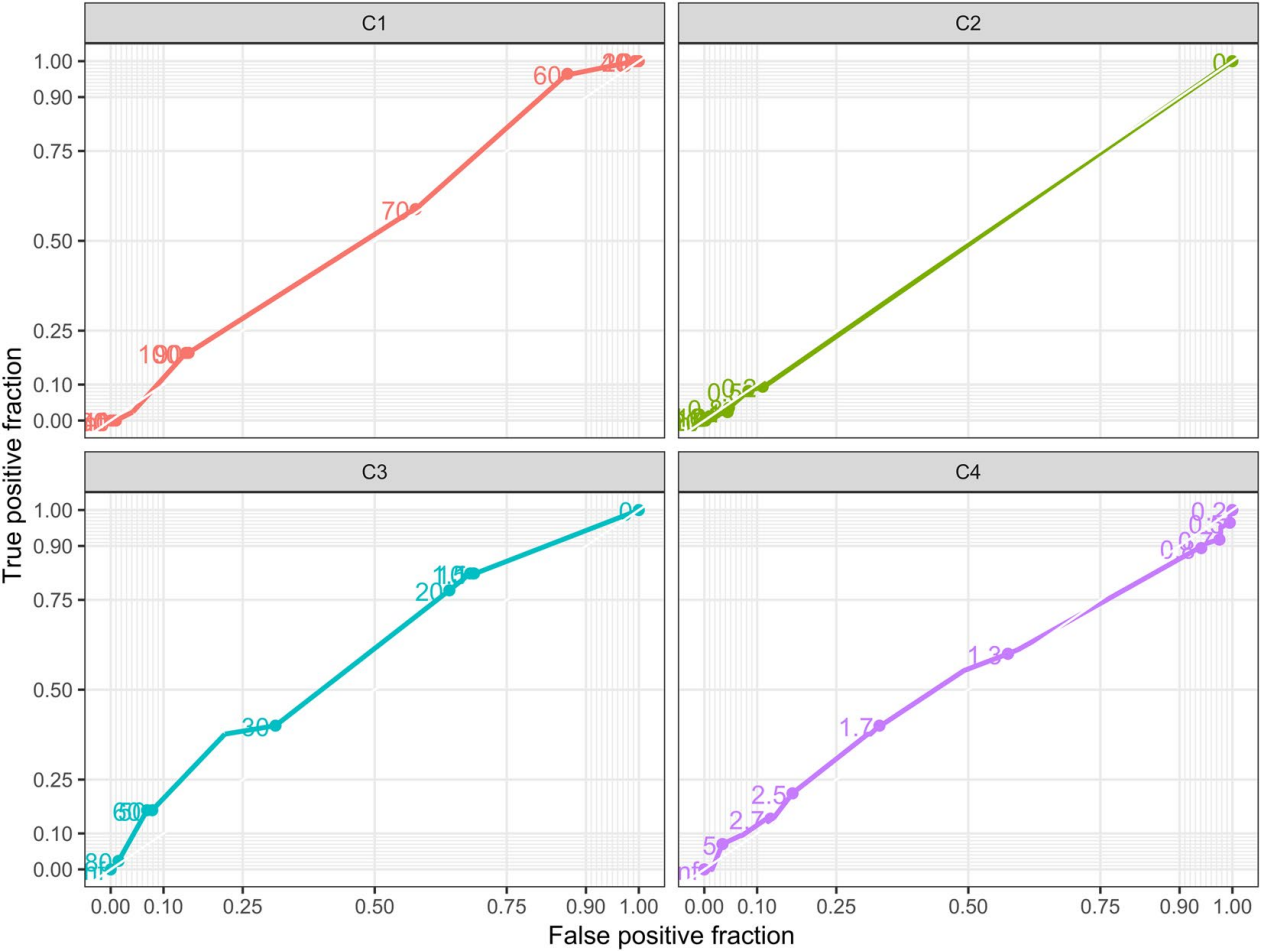
ROC curve for Echocardiographic Criteria LA/AO ≥ 1.8 and ECG criteria independent of left atrial indexed diameter z-score C1: P wave duration ≥ 110msec C2: P mitrale ≥ 40msec C3: Terminal negative P wave in lead V1 ≥ 40msec C4: P/PR segment ≥ 1.6.

Interestingly, the terminal negative P wave in V1 and increased LA/AO ratio appear in the two pairings that achieved fair agreement. In order to determine the underlying driving force behind these pairings, ROC curves were constructed (Figs. 2 and 3). ROC curves were also constructed for LA dimension z-scores. The corresponding areas under the curves (AUCs) demonstrate that of the selected ECG criteria, a terminal negative P wave in V1 is the most strongly correlated with the echocardiographic criteria, and can be considered the best indicator. However, it is important to note that at best, a terminal negative P wave in V1 is only marginally better than random (C3/EC1 AUC:0.580, C3/EC2 AUC:0.581) and the other ECG criteria investigated showed relatively similar AUCs ranging from 0.522–0.576 for left atrial indexed diameter z-score ≥2.0 and 0.452–0.538 for LA/AO ratio ≥1.8.

In our subgroup analysis, 263 patients were in the <1 year age group and 221 patients were ≥1 year age group. 8 Patients were excluded from this analysis due to missing data. When LA/AO ratio ≥ 1.8 was used in patients in the ≥1 year age group, a combination of both P mitrale and terminal negative P wave in lead V1 indicated moderate agreement (κ = 0.433, CI 0.186–0.680). The combination of all 3 criteria resulted in highest agreement at a P wave duration cut-off of 70 msec (κ = 0.516, CI 0.236–0.796). In the <1 year age group, when LA/AO ratio ≥ 1.8, a combination of both P mitrale and terminal negative P wave in lead V1 was not significant. The highest agreement in this age group was fair agreement seen at LA/AO ratio ≥ 1.6 and P wave duration cut-off of 100 msec (κ = 0.267, CI 0.073–0.461). In this age group, both 1) P wave duration and terminal negative P wave in lead V1 and 2) P wave duration and P/PR segment demonstrate a fair agreement with left atrial indexed diameter z-score when P wave duration cut-off is 70 msec, (κ = 0.267, CI 0.094–0.441 and κ = 0.238, CI 0.106–0.371, respectively.)

## Discussion

In this study, we evaluated the diagnostic value of various ECG criteria for LAE in the pediatric population compared to the gold-standard echocardiogram. To our knowledge, this is the first study in over 30 years to evaluate these criteria in the pediatric population. Our findings indicate that the presence of both a P mitrale ≥40 msec and terminal negative p-wave in lead V1 ≥ 40 msec in combination may corroborate a diagnosis of LAE. However, ECG criteria should not be used to diagnose LAE in the absence of an echocardiogram and findings should be considered in the context of clinical symptoms. Our post-hoc subgroup analysis identified that the agreement between the echocardiogram and ECG criteria was generally higher in the patients aged greater than one years old. P mitrale ≥40 msec, in particular, was associated with increased agreement in this age group and not patients aged less than one year old. There did not appear to be a relationship between P wave duration and age.

To the best of our knowledge, there have only been two studies in the literature that compare the use of ECGs with the gold-standard of echocardiogram for the diagnosis of LAE in the pediatric population. The relatively small sample size used in our study may have contributed to the low ROC value for all tested parameters, but given the smaller sample sizes in the other pediatric studies, this sample size is quite robust. Of note, this is the first study that attempts to validate previously unexplored ECG criteria including P mitrale and increased P/PR segment ratio criteria in ECGs in the pediatric population. Biancaniello *et al*. have used three criteria to diagnose LAE that have not been used in this study: (1) P wave amplitude ≥2.5 mm; (2) P wave duration > 0.08 seconds and (3) negative terminal deflection in V1 ≥ −1 mm^6^. Of these criteria, the most similar criteria to those used in the current study is P wave duration >0.08 seconds compared with 0.110 seconds used in their study. While the sensitivity for these individual criteria is not reported, the sensitivity for all criteria was 50% for the ECG in comparison with the echocardiogram. The sensitivity found in their study is higher than that for P wave duration criteria found in the current study, as expected due to the lower threshold value. This is consistent with the idea that a shorter P wave duration criterion would allow for detection of borderline cases of left atrial enlargement compared to the diagnostic criteria used in the present study. However, it is important to note that the results reported by Biancaniello *et al*. are limited as the specificity is not reported. Maok & Krongrad have used the same two criteria as Biancaniello *et al*.: P wave in any limb lead with a duration >0.08 seconds and negative terminal deflection in lead V1^6,7^. Using these criteria, they found that the sensitivity of ECG was 40% and the agreement between echocardiogram and ECG results was 38%. While the criteria used by Maok & Krongrad are different from the criteria used in our study, the reported sensitivity is comparable (50–77%) and the agreement between echocardiogram and ECG is slightly higher (38%). The lower agreement observed in our study may be due to our very young patient population (263 patients under the age of 1) as our subgroup analysis demonstrated increased agreement for patients aged ≥1 year old, reaching 52% for a combination with three ECG criteria and LA/AO ratio≥1.8. This suggests that the criteria used in this study may be more stringent for patients under the age of 1, which is further evidenced by high specificities (reaching 96% in our study). Overall, the aforementioned studies are in agreement with our findings, but may have overestimated both the agreement between ECG and echocardiogram and the sensitivity due to their modest sample sizes of 52 and 90 patients, respectively.

Although not currently validated in the pediatric population, these ECG criteria have long since been used in the adult population. Munuswamy *et al*. compared all 4 criteria used in our study to adult echocardiographic criteria and found very similar findings to that of the present study^14^. Similar to our study, they determined that a bimodal P wave with a duration of >40 msec in any lead had a sensitivity of 15% and a specificity of 100%. Additionally, their findings that P wave durations >110 msec had a sensitivity and specificity of 33% and 88%, and P:PR ratio >1.6 at 31% and 64% were also very much in line with our findings. However, their findings for the criteria involving a negative V1 P wave >40 msec varied greatly from the findings of the present study in that they reported a comparatively high sensitivity of 83% but similar specificity of 80%. Interestingly, a more recent study by Batra *et al*. investigating only V1 P wave >40 msec as a criterion for LAE in the adult population identified sensitivities and specificities of 54.4% and 57.14%^15^. The level of agreement between several criteria investigated in the present study and studies in the adult population may indicate greater similarities between populations than initially presumed. This agreement may therefore extend the generalizability of the extensive findings in the echocardiogram and ECG literature from the adult population to pediatric patients.

**Figure 4:**
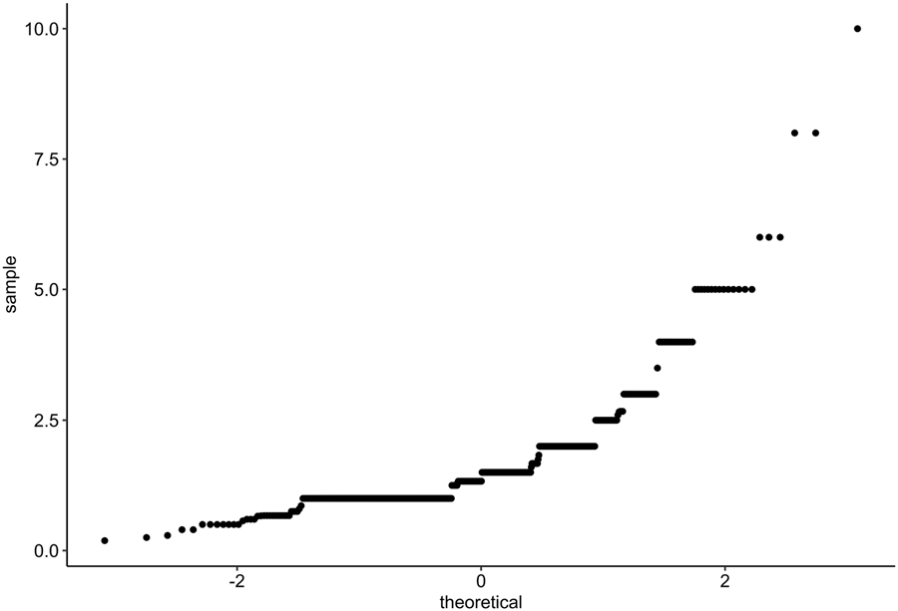
Q-Q plot for distribution of study participants by P/PR segment.

**Figure 5:**
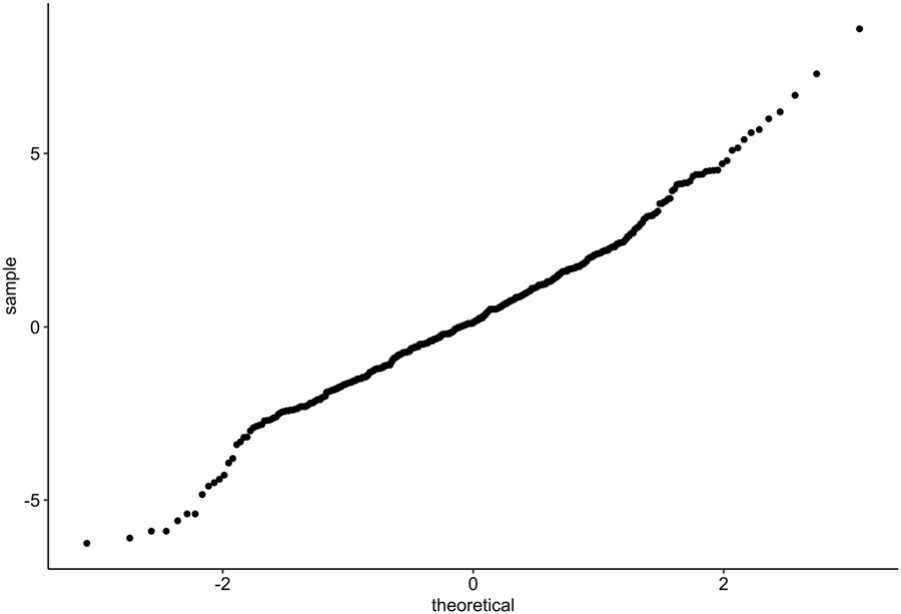
Q-Q plot for distribution of study participants by LA/AO ratio.

It is important to note that all the ROC analyses carried out in this paper are univariate ROC analyses. Although a multivariate ROC analysis of this data would be of great interest, this was not pursued for two reasons. Firstly, the most common multivariate ROC methods are based on the assumption of multivariate normal distribution for the characteristics of interest^16,17^. Secondly, a very large sample size is required to get the procedure to achieve a reasonable degree of sensitivity. In reviewing our data, simple histograms and Q-Q plots (Figs. 4 and 5) reveal that the characteristics of interest are skewed and are, therefore, not normally distributed which renders a multivariate ROC procedure based on the assumption of multivariate normality unsuitable. Moreover, as previously discussed, although the sample size in our study is relatively robust in the context of this field, it is not sufficiently large to support multivariate analyses. For these reasons, we have presented univariate ROC analyses performed separately on each characteristic of interest.

Our study had several limitations. Firstly, due to the retrospective nature of this study, participants could not be followed to correlate symptomatology, treatment, or treatment outcomes with observed ECG findings. Further studies are needed to determine how to apply findings in the context of the clinical presentation. Another potential limitation in our study is that left atrial volumes were not assessed in our study population. The American Society of Echocardiography’s guidelines recommend using the biplane area length method for performing left atrial volume (LAV) measurements by transthoracic echocardiography^3^. Use of two-dimensional LAV measurements have been shown to provide the most accurate measure of true left atrial size and are a reliable indicator of duration and severity of diastolic dysfunction in both adults and children. However, in current clinical practice at many hospitals nationwide, pediatric LAVs are not routinely measured nor interpreted for the purposes of treatment planning. Thus, our study sought to use left atrial indexed diameter Z-score ≥2.0 and elevated LA/AO ratio as indicators of LAE as these diagnostic factors are commonly reviewed by pediatric cardiologists. It is only more recently that we have started doing LA volume measurements routinely when the LA is enlarged. Additionally, we only gathered data from patients with LAE and we are not able to calculate the false positive rate of ECG criteria. Future studies should gather data from non-LAE control patients to estimate the baseline false positive rates of these criteria. Our post-hoc subgroup analysis demonstrated that age, even within the pediatric population, may modify the agreement between the echocardiogram and ECG. Future studies may want to further examine the relationship between tests for a wider distribution of ages, with a particular focus on P wave duration.

## Conclusions

This study, conducted in an acute care pediatric center serving a diverse and multicultural population, represents the largest retrospective chart review of its kind to date. We conclude that the proposed ECG criteria previously studied in adult populations have poor diagnostic value for LAE in the pediatric population when compared to echocardiographic investigations. Of the ECG criteria studied, P mitrale ≥40 msec and terminal negative P wave in lead V1 ≥ 40 msec may, in combination with the appropriate clinical presentation, be helpful in identifying patients with the greatest degree of disease burden and thus help in prioritization and resource allocation for echocardiographic investigations. In the case of a patient presenting with significant signs and symptoms suspicious for LAE, the absence of ECG criteria should not be considered a deterrent to further investigation.
